# Comparison of the effects of corneal and lacrimal gland denervation on the lacrimal functional unit of rats

**DOI:** 10.5935/0004-2749.20220008

**Published:** 2022

**Authors:** Jacqueline Ferreira Faustino Barros, Ariane Mirela Saranzo Sant’Ana, Lara Cristina Dias, Adriana de Andrade Batista Murashima, Lilian Eslaine Costa Mendes da Silva, Marina Zilio Fantucci, Eduardo Melani Rocha

**Affiliations:** 1 Department of Ophthalmology, Otorhinolaryngology, and Head and Neck Surgery, Faculdade de Medicina de Ribeirão Preto, Universidade de São Paulo, Ribeirão Preto, SP Brazil

**Keywords:** Cornea, Lacrimal functional unit, Lacrimal apparatus, Hypersensitivity, Wistar rats, Dry eye syndrome, Córnea, Unidade functional lacrimal, Aparelho lacrimal, Hipersensibilidade, Ratos Wistar, Síndrome do olho seco

## Abstract

**Purpose:**

This study aimed to compare the changes in the lacrimal functional unit in the following two models of neurogenic dry eye syndrome: sensory denervation of the cornea versus autonomic denervation of the lacrimal gland.

**Methods:**

The neural network supports the lacrimal functional unit. It can be divided into afferent (sensory) and efferent (autonomic) pathways and is affected by severe diseases that compromise the lacrimal functional unit. Male Wistar, 8-week-old rats were divided into the following three groups: 1) control naïve (n=16 animals); 2) autonomic denervation: where rats were subjected to right lacrimal gland nerve ablation and evaluated after 1 and 2 months (1M and 2M) after the procedure (n=7 animals per subgroup, autonomic denervation 1M and autonomic denervation 2M, respectively); 3) sensory denervation induced by 0.2% benzalkonium chloride eye drops, twice a day for 7 days in the right eye (n=10 animals). The corneal sensitivity was measured using the eye wipe test with capsaicin (10 µM). The quantitative real-time PCR was performed to compare the mRNA expressions of proinflammatory cytokines, such as Il-1β, Il-6, Tnf, Mmp9, in the cornea, trigeminal ganglion, and lacrimal gland. In addition, the mRNA of the promitotic factors in the lacrimal gland, such as Bmp7, Runx1, Runx3, Fgf10, and Smad1, was compared.

**Results:**

Sensory denervation induced corneal hyperalgesia (p=0.001). Sensory denervation and autonomic denervation increased the mRNA of proinflammatory cytokines in the cornea and lacrimal gland (p<0.05), but only sensory denervation increased the mRNA levels of Il-1β and Tnf in the trigeminal ganglion (p<0.05) compared with the control naïve.

**Conclusions:**

Autonomic denervation and sensory denervation models can have common features, such as inflammation of different parts of the lacrimal functional unit. However, hyperesthesia and inflammatory markers in the trigeminal ganglion because of sensory denervation and the expression of regenerative mediators in the lacrimal gland owing to autonomic denervation are the distinguishing features of these diseases that can be explored in future studies assessing dry eye syndrome secondary to neural damage of the lacrimal functional unit.

## Abstract (pt)

**Objetivo:**

O nosso objetivo neste estudo foi comparar as alterações na unidade funcional lacrimal em dois modelos de síndrome do olho seco neurogênica: desnervação sensorial da córnea versus desnervação autonômica da glândula lacrimal.

**Métodos:**

A rede neural é um importante suporte para a unidade funcional lacrimal. Pode ser dividido em vias aferentes (sensoriais) e eferentes (autonômicas), sujeitas a doenças graves que comprometem a unidade funcional lacrimal. Ratos Wistar machos, com 8 semanas de idade, foram divididos em três grupos: 1) Controle naïve (n=16 animais); 2) Desnervação autonômica: onde os ratos foram submetidos à ablação do nervo da glândula lacrimal direita e avaliados após um e dois meses (1 M a 2 M) do procedimento (n=7 animais por subgrupo, desnervação autonômica 1M e desnervação autonômica 2M, respectivamente); 3) Desnervação sensorial induzida por colírio a 0,2% de cloreto de benzalcônio, duas vezes ao dia por 7 dias no olho direito (n=10 animais). A sensibilidade da córnea foi medida pelo teste de movimento pata-olho com capsaicina (10 µM). A PCR quantitativa em tempo real foi aplicada para comparar a expressão relativa de mRNA de citocinas pró-inflamatórias: Il1b, Il6, Tnf, Mmp9, na córnea, gânglio trigêmio e glândula lacrimal. O mRNA dos agentes pró-mitóticos Bmp7, Runx1, Runx3, Fgf10 e Smad1 foram comparados na glândula lacrimal.

**Resultados:**

A desnervação sensorial induziu hiperalgesia da córnea (p=0,001). Desnervação sensorial e desnervação autonômica aumentaram o mRNA de citocinas pró-inflamatórias no córnea e glândula lacrimal (p<0,05), mas apenas desnervação sensorial aumentou o mRNA de Il1b e Tnf no gânglio trigêmio (p<0,05) quando comparado ao controle naïve.

**Conclusões:**

Os modelos de desnervação autonômica e desnervação sensorial podem ter características comuns, como inflamação de diferentes partes da unidade funcional lacrimal. No entanto, a hiperestesia e os marcadores inflamatórios no gânglio trigêmio de desnervação sensorial e a expressão de mediadores regenerativos na glândula lacrimal na desnervação autonômica são características que distinguem essas doenças, podendo ser investigadas em estudos futuros que abordam o olho seco secundário ao dano neural da unidade funcional lacrimal.

## INTRODUCTION

The sensory and autonomic neural network supports the ocular surface (OS), and therefore, the diseases that target the neural network can cause dry eye syndrome (DES) and ocular surface structural and functional disruptions^(1-3)^. The neural damage of the cornea (CO) sensory network (sensory or afferent denervation, SD) or the autonomic, efferent neural damage (AD) to the lacrimal gland (LG) have common features, such as aqueous tear deficiency, OS sensitivity and inflammation, and corneal epitheliopathy, as evidenced in studies regarding the lacrimal functional unit (LFU)^(4-10)^. Although clinical findings could overlap, and certain conditions affect both the afferent and efferent pathways, the characteristics that distinguish sensory and autonomic LFU damage are unknown^(9)^.

Notably, DES and OS disease affects millions of people worldwide, causing discomfort, visual impairment, eye integrity compromise, and being more frequently associated with other diseases^(11,12)^. We hypothesized that understanding the distinctive aspects of each condition that causes DES and OS disease could help identify more specific diagnostic and therapeutic modalities because even though currently there are several diagnostic tests and treatments for DES, they have low predictive value and efficacy^(13-15)^.

Notably, benzalkonium chloride (BAK), widely used as a preservative in topical eye medications, induces OS toxicity, and causes DES^(14,16,17)^. BAK topical use induces DES, keratitis, increased cytokine expression, inflammatory cell infiltration on the corneal and conjunctival tissues, and squamous metaplasia. Notably, mice models have evidenced these effects could propagate to the trigeminal ganglion (TG)^(16,18-20)^.

LG denervation has been widely used since the 1940s as a “cure” for epiphora and tearing caused by environmental factors. Current evidence indicates that neural damage to the exocrine glands, including the LG, in humans and other species is associated with autonomic dysfunction, local inflammation, and secretory impairment and plays a part in the DES mechanism, such as in Sjögren’s syndrome^(8,21-23)^.

This study evaluated the functional and molecular effects of DES caused by BAK (afferent or sensory denervation, SD) or LG nerve ablation (LG efferent or autonomic denervation, AD) in rats. We hypothesized that SD and AD have distinct mechanistic features, despite their similar clinical presentations. Moreover, a deeper investigation into the physiopathology of SD and AD diseases would facilitate better diagnostic, predictive, and therapeutic approaches. The study objectives were to compare the SD and AD models with control rats in terms of tear flow, corneal sensitivity triggered by capsaicin, and the mRNA expression of inflammatory cytokines in CO, LG, and TG and that of the tissue repair mediators in the LG.

## METHODS

### Animals and study design

All experimental procedures adhered to the ARVO Statement for the Use of Animals in Ophthalmic and Vision Research and were approved by the committee for animal use at the Ribeirao Preto School of Medicine, University of Sao Paulo (Ribeirão Preto, SP, Brazil) (Protocol 109/2008).

Wistar male 8-week-old rats, weighing 220-250 g obtained from the Animal Breeding Center of the Ribeirao Preto Medical School, University of Sao Paulo, were divided into three groups, with the AD group subdivided further into two based on the endpoints. Hence, overall, the following four groups were analyzed:

Sensory denervation (SD): The rats (n=10 animals) received 5 µL of 0.2% BAK twice daily for 7 days in the right eye. BAK was obtained (Fluka Analytical, Sigma-Aldrich Brazil Ltda. COTIA, SP, BRAZIL), diluted in phosphate buffer at 25 ºC and pH 7.2. The procedure began with animal immobilization, instillation of a drop of 0.2% BAK in the right eye, and after 10 seconds, allowing the drug to spread, each rat was returned to its cage.

Autonomic denervation (AD): The rats were subjected to surgical denervation of the exorbital LG, and the outcomes were evaluated after 1 and 2 months (n=7 animals per group).

LG denervation was performed as follows: Under intramuscular anesthesia with xylazine (Laboratorio Callier S.A., Barcelona, Spain [15 mg/kg]) and ketamine (União Química Farmacêutica S.A, Embu-Guaçu, SP, Brazil [150 mg/kg]), an aseptic skin incision was performed between the eye and the ear on the right side. The extraorbital LG was identified, the LG nerve branch detected, isolated from the vascular branches, resected, and repositioned, avoiding contact between the cuts, based on techniques previously described^(6,22)^. After homeostatic control, the surgical wound was closed with cyanoacrylate glue (Locite, Henkel Ltda, Diadema, SP, Brazil) and covered with a single, 5-mm application of antibiotic and anti-inflammatory ointment (Cylocort, União Química Farmacêutica Nacional S.A, Brasilia, DF, Brazil).

The two AD subgroups were evaluated 1 and 2 months after the procedure (n=7 animals per subgroup, AD 1M and AD 2M).

Control group (CG): The group without any intervention (naïve) was included for comparison and was evaluated after 5 weeks of housing in the same vivarium (n=16 animals). All rats were housed in cages at a nearly constant temperature (23 ± 2 ºC) in light-dark cycles of 12 h. Animals had ad libitum access to standard rodent chow and water.

### Eye wipe test

At the end of the experimental period for each group, namely 7 days for the SD group, 1 month and 2 months for the AD group, and 5 weeks for the CG, the rats were subjected to the eye wipe test in response to capsaicin (CAP) to investigate the CO sensitivity. After acclimation of the animals to Plexiglas chambers for 1 hour, the right eye of all rats were instilled with 20 µL of 10 µM CAP diluted in PBS at pH 7.2 and 25 ºC (Sigma-Aldrich Brazil Ltda., Cotia, SP, BRAZIL).

The eye wipe behavior was recorded using a digital camera (DSC-W5, Sony, Japan) for 3 min after the instillation of CAP. Eye wipe movements (EWT) recorded for 3 min, starting after the CAP eye drop instillation, were analyzed based on the digital recording of each rat by a masked observer with an iMac computer (Apple Inc, Cupertino, CA, USA) and compared with the CG.

### Clinical evaluation

Furthermore, to investigate the effects of SD and AD on the CO and tear flow, the animals were evaluated under general anesthesia after an intraperitoneal injection of ketamine (5 mg/100 g body weight) (União Química Farmacêutica S.A, Embu-Guaçu, SP, Brazil) and xylazine (2 mg/100 g body weight) (Laboratorio Callier S.A., Barcelona, Spain) to collect the following observations:

Corneal epithelial integrity was evaluated using slit-lamp after 2% sodium fluorescein dye staining. The punctate keratitis was graded from 0 to 15, as previously described^(24)^. In addition, the presence of epithelial defects was examined.

Tear flow was measured in millimeters using the red phenol thread (RPT) for 30 seconds, and the values obtained were compared among the groups (Showa Yakuhin Kako Co; Ltd, Tokyo, Japan & Menicon USA Inc., Clovis, CA, USA).

Notably, the duration of the experimental period was different for SD, AD, and CG after the rats completed 8 weeks of life, and they were housed in the experimental vivarium. It was 7 days for the SD group during the BAK use, 1 and 2 months for the AD group (counting from the day of LG nerve ablation), and 5 weeks for the CG (counting from the day they were relocated to the experimental vivarium). The reason for the different durations was based on observations from previously published works and pilot studies that indicated the requisite duration for obtaining the ocular manifestations^(16,22)^. Notably, longer periods of BAK would induce excessive toxicity, and interrupting its use would revert the treatment effects ^(18,20)^. Moreover, earlier observation of surgical denervation would only reveal the inflammatory effects of the procedure. The experimental period for the CG was selected as an intermediary period to SD and 2-month AD^(22)^.

### Quantitative real-time PCR

After in vivo observations, the animals were euthanized using ketamine (5 mg/100 g body weight) (União Química Farmacêutica S.A, Embu-Guaçu, SP, Brazil), xylazine (2 mg/100 g body weight) (Laboratorio Callier S.A., Barcelona, Spain), and thiopental sodium (1000 mg/kg) (Laboratório Cristália, São Paulo, SP, Brazil). The CO, LG, and TG tissues were harvested from the right side of the rats of all three groups and imbedded in RNA stabilization solution (RNAlater Solution, Ambion, Waltham, MA, USA) and stored at -80 ºC until RNA extraction, quantification, quality evaluation, and quantitative real-time PCR (qPCR) analysis. The relative expressions of the mRNA of proinflammatory cytokines, such as Il1β, Il-6, Tnf, and Mmp9, in the LG, CO, and TG samples obtained from all three study groups were compared. In addition, the relative mRNA expressions of the tissue repair elements in the LG, namely the Bmp7, Runx1, Runx3, Fgf10, and Smad1, were compared among the three groups.

The qPCR was performed using hydrolysis probes (Applied Biosystems, Carlsbad, CA, USA). Total RNA samples were extracted from the tissues by using RNeasy Mini Kit (Qiagen, Germantown, MD, USA), according to the manufacturer’s instructions, and was quantified using a NanoDrop 2000c spectrophotometer (Thermo Scientific, Wilmington, DE, USA).

Samples containing 500 ng of total RNA of CO tissue, 1000 ng of total RNA of LG tissue, and 350 ng of TG of AD group and 150 ng of SD group were used to synthesize the cDNA with the QuantiTect Reverse Transcription Kit (Qiagen, Germantown, MD, USA) in the ProFlex PCR System (Applied Biosystems, Carlsbad, CA, USA). The qPCR was performed using the ViiA7 Real-Time PCR System (Applied Biosystems, Carlsbad, CA, USA). The following hydrolysis probes were used in this study: Rn.PT 5838028824 (*Il-1*β), Rn.PT 5813840513 (*Il-6*), Rn.PT 5811142874 (*Tnf*), Rn.PT 587383134 (*Mmp9*), Rn.PT 5810180444 (*Bmp7*), Rn.PT 5810814634 (*Fgf10*), Rn.PT 589220704.g (β-actin) (all these from IDT); Rn00565555_m1 (*Smad1*), Rn00569082_m1 (*Runx1*), Rn00590466_m1 (*Runx3*) (Applied Biosystems, Carlsbad, CA, USA). Each amplification reaction was performed in duplicate with 5.5 µL of QuantiNova Probe PCR Kit (Qiagen, Germantown, MD, USA), 0.5 µL of hydrolysis probe, and 4.5 µL of 1:4 dilution of the cDNA in a total volume of 10 µL. The cycles for real-time PCR were as follows: one cycle of 95 ºC for 2 minutes, 50 cycles of 5 seconds at 95 ºC and 19 seconds at 60 ºC.

The relative quantification was determined using the Thermo Fisher Cloud Software, RQ version 3.7 (Life Technologies Corporation, Carlsbad, CA, USA).

### Statistical analysis

The software GraphPad Prism 8.0 (GraphPad Software, San Diego, CA, USA) was used to obtain descriptive statistics and compare the response to the EWT, RPT tests, and laboratory exams among the SD, AD, and CG groups by using the non-parametric, one-tailed, and Mann-Whitney U statistical tests. The level of significance was set at *p*<0.05.

## RESULTS

**Figure 1:**
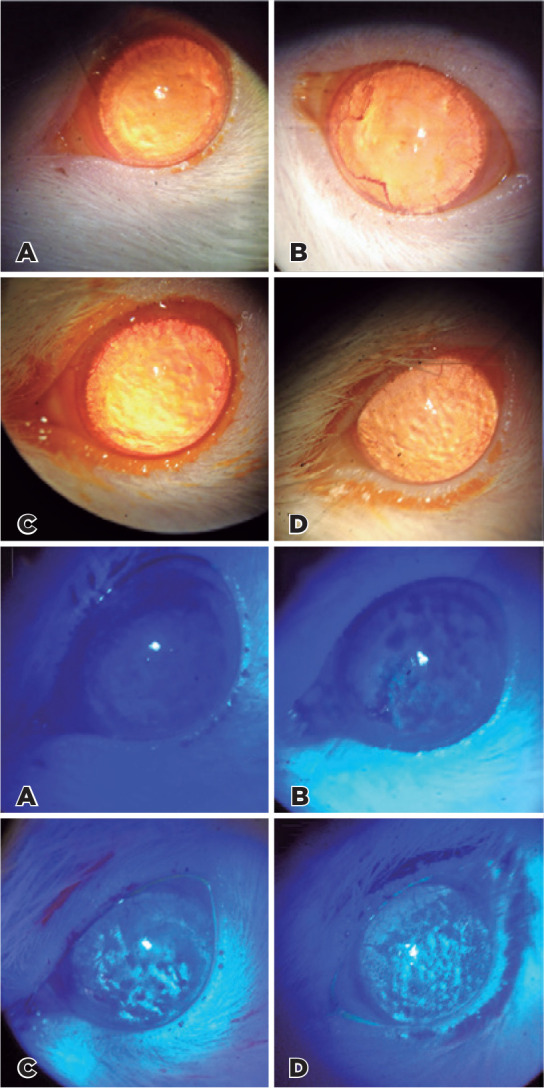
Corneal slit-lamp examination: 1) the white light with sodium fluorescein dye staining, all groups exhibited mild keratitis and neovascularization, albeit non-significant (*p*>0.05). A) CG group; B) SD group; C) AD 1M; D) AD 2M. 2) Corneal slit-lamp examination with the cobalt blue light with sodium fluorescein dye staining, all groups exhibited mild keratitis, albeit non-significant (*p*>0.05). A) CG group; B) SD group; C) AD 1M; D) AD 2M. (CG: 10 rats, SD: 10 rats, AD 1M: 7 rats, AD 2M: 7 rats).

Upon corneal slit-lamp examination, all groups revealed mild keratitis and neovascularization (Figures 1A and B).

**Figure 2:**
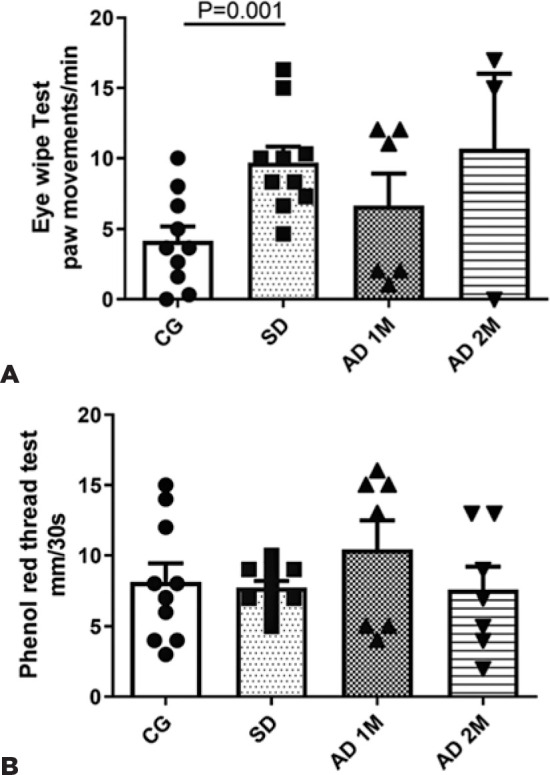
A) The results of eye wipe test (EWT) based on the paw movements per minute for 3 minutes, a minute after receiving an eye drop of capsaicin 10 _µ_M (CG: 10 rats, SD: 10 rats, AD 1M: 6 rats, AD 2M: 3 rats). In this test, the SD exhibited more frequent EWT, compatible with hyperalgesia (*p*=0.0010). B) Phenol red thread test (PRT) measured in millimeters of tears wetting the cotton thread in 30 seconds (CG: 10 rats, SD: 10 rats, AD 1M: 7 rats, AD 2M: 7 rats), where SD group and AD 2M subgroup exhibited lower mean tear flow, although non-significant (*p*>0.05). Statistical analysis was performed using Mann-Whitney U test.

Regarding EWT, the SD group exhibited a higher frequency of paw eye wipe movements after CAP sensitization compared with CG (p=0.001), indicating hyperalgesia after the BAK-induced corneal nerve damage. The AD 1M and 2M subgroups did not exhibit significant differences regarding the EWT compared with the CG (Figure 2A and Table 1).

**Table 1:** Summary of the clinical and molecular results of the autonomic neural damage (AD) of the LG (1 and 2 months) and sensory nerve damage (SD) of the cornea (CO), compared with the control group (CG). _↑_ means increase compared with the CG

	Control group (CG)	Ablation of LG nerve (AD)	Denervation of the CO (SD)
Number of rats	16	7	10
Experimental time	5 weeks	1 month and 2 months	7 days
Corneal sensitivity			↑
Cytokines cornea (CO)		↑	↑
Cytokines trigeminal ganglion (TG)			↑
Promitotic mediators lacrimal gland (LG)		↑	↑

The tear flow measured using the Phenol red thread test (PRT) revealed lower median levels in SD and AD 2M groups, but not significantly different from CG (Figure 2B and Table 1).

**Figure 3:**
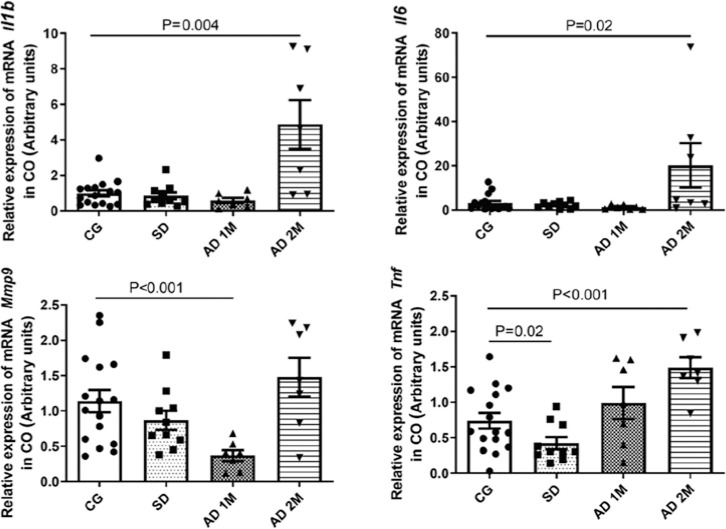
Comparative analysis of mRNA expressions of proinflammatory cytokines Il-1_β_, Il-6, Mmp9, and Tnf through qPCR in the CO (expressed in arbitrary units, normalized using _β_-actin mRNA). The SD group exhibited lower relative mRNA expression of Tnf (*p*=0.02). The AD 1M group had lower relative mRNA expression of Mmp9 (*p*<0.001). The AD 2M exhibited higher mRNA expressions of Il-1_β_, Il-6, and Tnf (*p*=0.004, *p*=0.02, and *p*<0.001, respectively) (CG: 16 rats, SD: 10 rats, AD 1M: 7 rats, AD 2M: 7 rats). Statistical analysis was performed using Mann-Whitney U test.

The qPCR analysis of proinflammatory cytokine mRNA revealed that SD decreased the Tnf mRNA in the CO (p=0.02). Moreover, AD decreased the Mmp9 expression after 1 month (p<0.001), and increased the expressions of Il-1β, Il-6, and Tnf after 2 months (p=0.004, p=0.02, and p<0.001, respectively) (Figure 3 and Table 2).

**Table 2:** Summary of the qRT-PCR results of proinflammatory cytokines and promitotic elements in response to autonomic neural damage (AD) of the LG (1 and 2 months) and sensory nerve damage (SD) of the cornea (CO), compared with the control group (CG). _↑_ means significant increase compared with the CG and _↓_ means significant decrease compared with the CG

Model		AD 1M			AD 2M			SD	
Tissue	CO		LG	CO		LG	CO	TG	LG
Il-1**β**				**↑**				**↑**	**↑**
Il-6				**↑**					**↑**
Mmp9	**↓**		**↑**			**↑**			**↓**
Tnf							**↓**	**↑**	
Tissue		LG			LG			LG	
Bmp7			**↓**			**↓**			
Fgf10			**↓**			**↓**			
Runx1									**↓**
Runx3			**↑**						**↓**
Smad1									**↓**

The abbreviations are associated with the mRNA sequences of the following cytokines: Il-1β= interleukin 1β; Il-6: interleukin-6; Mmp9= matrix metalloproteinase-9, Tnf= tumor necrosis factor; and promitotic molecules: Bmp7= bone morphogenetic protein-7; Fgf10= fibroblast growth factor 10; Runx1= runt-related transcription factor-1; Runx1= runt-related transcription factor.

**Figure 4:**
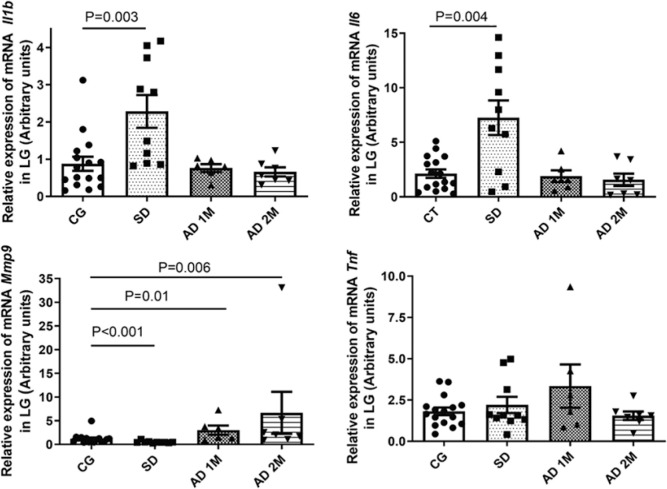
Comparative analysis of mRNA expression of proinflammatory cytokines Il-1_β_, Il-6, Mmp9, and Tnf through qPCR in the LG (expressed in arbitrary units, normalized using _β_-actin mRNA). The SD group exhibited higher expressions of Il-1_β_ and Il-6 (*p*=0.003 and *p*=0.004, respectively), and lower expression of Mmp9 compared with the CG (*p*<0.001). AD 1M and 2M exhibited higher expression of Mmp9 (*p*=0.01 and *p*=0.006, respectively) (CG: 16 rats, SD: 10 rats, AD 1M: 6 rats, AD 2M: 7 rats). Statistical analysis was performed using Mann-Whitney U test.

Furthermore, in the LG, SD increased the mRNA expression of the proinflammatory cytokines Il-1β and Il-6 (p=0.003 and p=0.004, respectively) and reduced the mRNA expression of Mmp9 (p<0.001). AD increased the mRNA expression of Mmp9 after 1 and 2 months (p=0.01 and p=0.006, respectively) (Figure 4 and Table 2).

**Figure 5:**
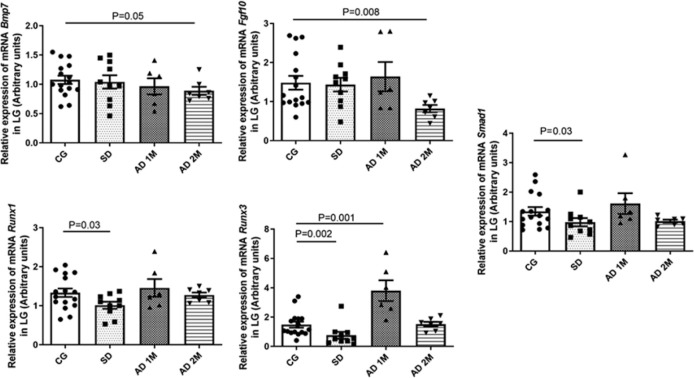
Comparative analysis of the mRNA expressions of the promitotic elements, namely Bmp7, Fgf10, Runx1, Runx3, and Smad1, in the LG through qRT-PCR (arbitrary units, normalized using _β_-actin mRNA). SD group exhibited lower expression of Runx1, Runx3, and Smad1 (*p*=0.03, *p* 0.002, and *p*=0.03). AD 1M exhibited higher expression of Runx3 (*p*=0.001). AD 2M exhibited lower mRNA expression of Bmp7 and Fgf10 (*p*=0.05 and *p*=0.008, respectively) (CG: 16 rats, SD: 10 rats, AD 1M: 6 rats, AD 2M: 7 rats). Mann-Whitney U test was used for statistical analysis.

Upon evaluation of the promitotic mediators in the LG, the SD group exhibited low mRNA expressions of Runx1, Runx3, and Smad1 (p=0.03, p=0.002, and p=0.03, respectively). Moreover, the LG of the AD 1M subgroup exhibited higher mRNA expression of Runx3 (p=0.001) and that of AD 2M revealed lower mRNA expression of Bmp7 and Fgf10 (p=0.05, p=0.008) (Figure 5 and Table 2).

**Figure 6:**
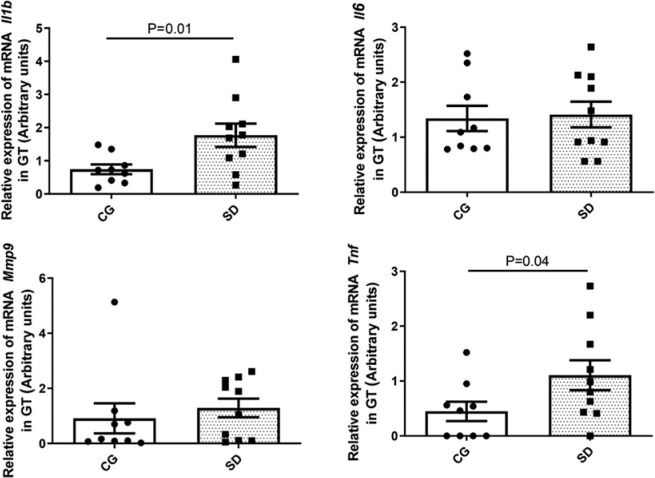
Comparative analysis of the mRNA expression of proinflammatory cytokines Il-1_β_, Il-6, Mmp9, and Tnf through qPCR in the TG (expressed in arbitrary units, normalized using _β_-actin and GAPDH). SD group exhibited higher levels of Il-1_β_ and Tnf (*p*=0.01 and *p*=0.04, respectively) (CG: 9 rats, SD: 10 rats). Mann-Whitney U test was used for statistical analysis.

The proinflammatory cytokine mRNA in the TG revealed the following profile: SD group exhibited higher mRNA expression levels of Il-1β and Tnf (p=0.01 and p=0.04) (Figure 6 and Table 2). Nevertheless, the TG of the AD 1M and 2M subgroups did not reveal any changes related to the proinflammatory cytokines (data not shown).

## DISCUSSION

The present work revealed that SD of the CO and AD of the LG induced inflammatory changes in the CO and LG, but only SD increased the proinflammatory markers in the TG, which was accordant with the manifestation of hyperesthesia, in response to CAP in the CO, in the SD model. By contrast, AD of the LG induced the expression of the promitotic Runx3 in the LG. These observations indicate that neural damage promotes a proinflammatory deviation, extending to other organs of the LFU.

The failure in inducing low tear flow with BAK, in contrast to previous studies that noted impaired tear flow, increased epitheliopathy, and corneal hypersensitivity, could be explained based on the low sensitivity and poor correlation among the DES methods, influenced by external factors like anesthesia and environmental humidity^(16,18,25,26)^.

The SD of the CO induced by BAK increased the expression of proinflammatory mediators, not just in the CO, as evidenced previously in mice, but also in the LG^(20,27,28)^. Moreover, this finding was concordant with previous studies, which observed that topical BAK use and alkali burn of the CO caused TG inflammation in mice^(20,29)^.

The hyperesthesia, demonstrated by the higher numbers of EWT in response to CAP, observed in the SD model and not in the AD model, accord with the mechanism of trigeminal pain because of persistent inflammation^(1,20)^. Furthermore, AD through LG nerve ablation at 1 and 2 months preserved the tear flow and normal CO sensitivity to CAP in the rats, and this was concordant with a previous work that used saporin toxin to induce LG denervation and observed CO hypersensitivity to menthol but not to CAP^(8)^.

The AD of the LG induced few changes in the LFU after 1 month, evidenced by a modest increase in the proinflammatory Mmp9 and an increase in the promitotic Runx3, suggesting an attempt to regenerate the LG tissue after the initial period of surgical manipulation. However, the AD of the LG after 2 months revealed an increase in all the proinflammatory cytokines tested in the CO, even more than the SD, with Mmp9 continuing to rise in the LG. Unlike the SD model presented here or mentioned above, the AD did not alter the mRNA cytokine profile in the TG, confirming the preservation of corneal sensitivity.

Notably, the promitotic mediators in the LG increased only in the AD model, suggesting that regenerative mechanisms of the LG are at work after LG denervation but return to the baseline in the second month. These findings are concordant with a previous work, which observed that parasympathetic disruption of the LG perpetuated the expression of proinflammatory cytokines and pro-apoptotic mediators for more than 2 months and impaired the capacity of constitutive proteins synthesis^(22)^. Notably, the inflammation of LG did not affect the neural network but impaired the tear secretion process mediated by the autonomic network, as observed in mice models of autoimmunity and in vitro studies^(30,31)^. Nevertheless, the preservation of the tear flow and CO observed in the AD model could possibly be due to the support of the other LG (i.e., infraand intraorbital) in the rat^(32)^.

The rationale of the present work is to analyze the mechanisms of neural injury of the LFU. In addition, we intended to distinguish the manifestations of SD and AD. In clinical practice, several diseases can disrupt the sensory or the autonomic motor network that supports the LFU, involving the CO and LG. These diseases include diabetes mellitus, herpes zoster, herpes simplex keratitis, Hansen disease, surgeries, trauma, and other diseases that can cause oculomotor, trigeminal, or facial nerve neuropathy. Notably, the limitations in identifying the precise topographic and molecular mechanisms in the clinical setting are associated with the lack of non-invasive methods and case presentations, which are frequently overlapped by severe complications like tissue ulceration and secondary infection.

In conclusion, AD and SD models have common features like inflammation of various LFU parts. However, hyperesthesia and inflammatory markers in the TG of the SD model and the expression of regenerative mediators in LG of the AD model are the distinguishing features of these diseases that can be explored by future studies concerning DES secondary to neural damage of the LFU.
